# Teicoplanin as an Effective Alternative to Vancomycin for Treatment of MRSA Infection in Chinese Population: A Meta-analysis of Randomized Controlled Trials

**DOI:** 10.1371/journal.pone.0079782

**Published:** 2013-11-18

**Authors:** Yang Peng, Xiaohua Ye, Ying Li, Tao Bu, Xiaofeng Chen, Jiaqi Bi, Junli Zhou, Zhenjiang Yao

**Affiliations:** Department of Epidemiology and Health Statistics, Guangdong Key Laboratory of Molecular Epidemiology, Guangdong Pharmaceutical University, Guangzhou, China; University of Iowa Carver College of Medicine, United States of America

## Abstract

**Objective:**

To evaluate whether teicoplanin could be an alternative to vancomycin for treatment of MRSA infection in Chinese population using a meta-analysis in randomized controlled trials.

**Methods:**

The following databases were searched: Chinese Biomedical Literature database (CBM), Chinese Journal Full-text database (CNKI), Wanfang database, Medline database, Ovid database and Cochrane Library. Articles published from 2002 to 2013 that studied teicoplanin in comparison to vancomycin in the treatment of MRSA infected patients were collected. Overall effects, publishing bias analysis and sensitivity analysis on clinical cure rate, microbiologic eradication rate and adverse events rate were performed by using Review Manager 5.2 and Stata 11.0 softwares.

**Results:**

Twelve articles met entry criteria. There was no statistically significant difference between the two groups regarding the clinical cure rate (risk ratio [RR], teicoplanin vs vancomycin, 0.94; 95% CI, 0.74∼1.19; P = 0.60), microbiological cure rate (risk ratio [RR], teicoplanin vs vancomycin, 0.99; 95% CI, 0.91∼1.07; P = 0.74) and adverse event rate (risk ratio [RR], teicoplanin vs vancomycin, 0.86; 95% CI, 0.40∼1.84; P = 0.70).

**Conclusions:**

The meta-analysis results indicate that the two therapies are similar in both efficacy and safety, thus teicoplanin can act as an effective alternative to vancomycin for treating patients infected by MRSA.

## Introduction

Methicillin-resistant *Staphylococcus aureus* (MRSA) has spread all over the world. It has caused serious health consequences since it was first identified in 1961 [1]. A study showed that the number of patients infected by MRSA has more than doubled, from 127000 in 1999 to 278000 in 2005 in US. In the same period, the number of deaths increased from 11000 to 17000 in USA [2]. Another study reported that MRSA has caused about 94360 severe infections and 18650 in-hospital deaths in 2005 in USA [3]. There were more reports about increasing incidence of MRSA from other countries [4], [5]. MRSA infection in China is also very severe. According to a report from Chinese National Healthcare Safety Network, MRSA accounts for 53% of *Staphylococcus aureus*(*S.aureus*), which occupies the largest proportion of the overall gram-positive bacteria [6].

Vancomycin, one of the most common glycopeptides, has been used as a golden standard for the treatment of patients with MRSA infection, especially for those with life-threatening disease [7]. Nevertheless, the role of vancomycin in the treatment of MRSA has been questioned and debated due to the spread of 1) vancomycin-intermediate *S. aureu*s (VISA), first described in a Japanese child who was clinically unresponsive to vancomycin [8], and 2) vancomycin-resistant *S. aureus* (VRSA), first reported in an American patient who was naturally resistant to vancomycin [9]. To date, both VISA and VRSA have been reported in many countries, including China [10]–[14]. Moreover, the vancomycin-resistant strains have caused the elevated minimum inhibition concentrations (MICs) in vancomycin, a phenomenon known as MIC creep. Although their MICs are still within the susceptible range, the isolated strains have accompanied with an alarming rise of mortality rate [15], [16]. The MIC creep was also detected in China [17], [18]. Therefore, finding an alternative therapeutic medicine is of great urgency. Teicoplanin, also a glycopeptides, is commonly used to treat β-lactam-resistant gram-positive pathogens, including MRSA. It shows a great therapeutic effect to MRSA. In China, several studies have been done in comparing the clinical efficacy and safety of the two antibiotics. Various experimental designs have been used, various participants were involved, and their conclusions were not always in consistence due to the limited sample size, participants, treatment length, among other factors. Hence, it is necessary to conduct a meta-analysis comprehensively comparing the efficacy and safety of the two antibiotics in the treatment of Chinese MRSA patients in order to explore whether teicoplanin can be used as an alternative to vancomycin in the treatment of patients infected by MRSA.

## Materials and Methods

### Literature Database

The meta-analysis followed the PRISMA guidelines [19]. The checklist detail was listed in Checklist S1. Major electronic databases were systematically searched. They were Chinese Biomedical Literature Database (CBM), China National Knowledge Infrastructure (CNKI) database, Chinese VIP database, Chinese Wan fang database, Medline database, Ovid database and Cocharane Library. Key words used for search were: (“MRSA” or “methicillin resistant Staphylococcus aureus”), (“vancomycin”), (“teicoplanin”) and (“RCTs” or “randomized controlled trials”). All papers, published from January 2002 to May 2013, on the comparison between vancomycin and teicoplanin in the treatment of MRSA in Chinese population were included. No language restrictions were applied.

### Inclusion and Exclusion Criteria

To be included in this Meta-analysis, studies must meet the following criteria: (a) RCTs; (b) the subjects were proven MRSA-infected Chinese patients; (c) the two parallel groups were treated with vancomycin and teicoplanin, respectively; (d) the outcome indicators included at least one of the followings: clinical cure rate, microbiological eradication rate and adverse event rate. Studies were excluded when they were: (a) missing baseline information, or a lack of medical information or statistical differences of baseline information were detected; (b) duplicate of previous publications; (c) reviews, letters, editorial articles or meta-analyses; (d) changing antibiotics during treatments.

### Data Extraction

Data from the published studies were extracted independently by two reviewers. For each study, the following characteristics were collected: the first author, year of publication, location, number of subjects, age, gender ratio, and outcome of indicators were also included. In case of conflicting evaluations, the disagreements were resolved by discussion among the whole group members.

### Quality Assessment of Included Studies

Two reviewers independently assessed the quality of included studies according to the Jadad standards [20]. The overall scores range from 0 to 5. Scores of 0∼2 and 3∼5 were regarded as low and high scores, respectively. Disagreements were also settled down by discussion among authors.

### Statistical Analysis

Statistical heterogeneities, included clinical cure rate, microbiological rate and adverse events rate, were estimated using Chi-square based Q statistic with a P-value <0.1 as statistically significant heterogeneity [21]. We also quantified the effect of heterogeneity by using the I^2^ test (ranges from 0 to 100%). A significant Chi-square based Q test with P<0.1 or I^2^>50% [22] indicated that heterogeneity among studies existed. The random effects model (DerSimonian Laird method [23]) was conducted for meta-analysis. Otherwise, the fixed effect model (Mantel-Haenszel method [24]) was used. Risk Ratios (RRs) with 95% confidence intervals (CIs) were calculated to assess the clinical cure rate, microbiological rate and adverse events rate of the two antibiotics, respectively. The funnel plots, Begger’s rank correlation test [25] and Egger’s linear regression test [26] were introduced to assess the publication biases, with P<0.1 indicating potential bias. In addition, sensitivity analysis was applied to assess the influence of each individual study. Forest plots and funnel plots were provided by the Review Manager 5.2 software and the Begger’s rank correlation test, Egger’s linear regression test and sensitivity analysis were conducted by the STATA (Version 11.0).

## Results

### Characteristics of the Eligible Studies

**Figure 1:**
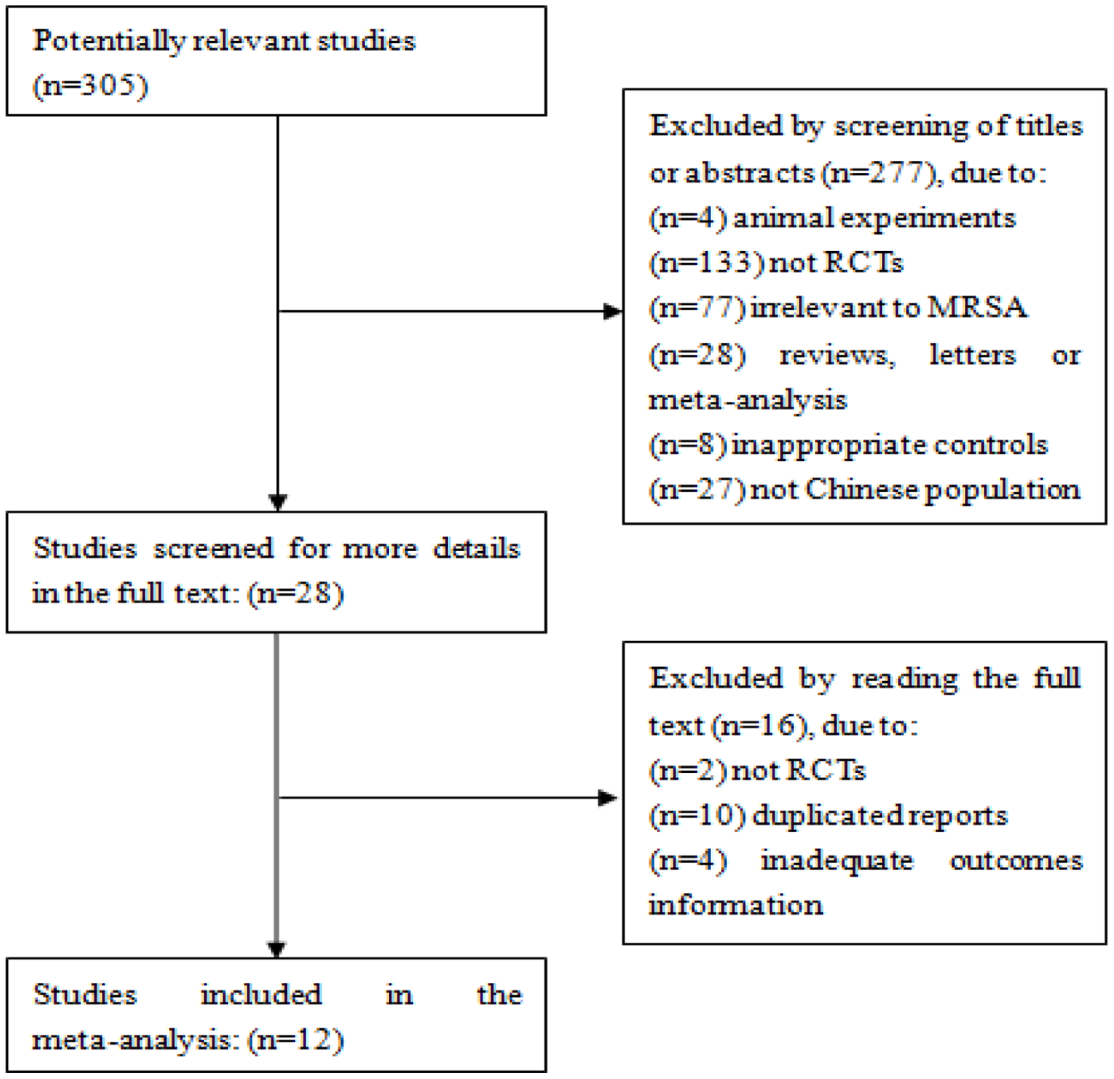
Flow diagram of the selection process of the included studies.

The process of selecting studies for the meta-analysis is shown in Figure 1. Twelve randomized controlled trials were included in our meta-analysis [27]–[38]. Among them, eight studies containing the clinical trial rate [28], [31], [33]–[38], nine studies containing the microbiological eradication rate [27]–[34], [37] and four studies involved the adverse event rate [31], [33], [34], [38]. The main characteristics of the included studies were summarized in Table 1. Subjects in three studies were gram-positive bacteria infected patients with no exact age and gender information but the authors declared that there were no statistical differences of age and gender ratio between the two groups. The Jadad scores of each study listed in Table 2.

**Table 1:** Main characteristics of included studies.

Study	Year	T/V^a^	Locations	Age(T/V)	Gender(M/F)	Outcomes
ZhaoWF [27]	2003	NP^b^	Jiangsu	NP	NP	ME^c^
Zhu HL [28]	2003	29/35	Shanghai	81.4/79.5	61/3	CC^d^,MEand AE^e^
ZhangTT [29]	2003	NP	Guangdong	NP	NP	ME
Xie JJ [30]	2006	29/21	Hunan	NP	NP	ME
Sun XX [31]	2009	17/19	Shandong	64/62	25/11	CC, ME and AE
Zu YN [32]	2010	NP	Henan	NP	NP	ME
Dong L [33]	2010	28/32	Shanghai	62.25/53.15	45/15	CC,ME and AE
Li XB [34]	2011	32/30	Sichuan	70.7/68.4	41/21	CC,ME and AE
Guo ZY [35]	2011	30/30	Guangdong	48∼68*	38/22	CC
Wang H [36]	2011	7/17	Shanxi	51∼88*	18/6	CC
Zhao N [37]	2013	32/32	Shandong	73.1*	39/16	CC and ME
Wang F [38]	2013	5/7	Jiangsu	24∼69*	8/4	CC and AE

a vancomycin/teicoplanin.

b Not Provided.

c Microbiological Eradiation.

d Clinical Cure.

e Adverse Events.

* The combined data of the two groups.

**Table 2:** Methodical assessment of included studies.

Study	Randomization	Details ofrandomization	Double-blind	Details of double-blind	Details ofdrop out	Jadad Score
ZhaoWF [27]	Y	N	N	N	Y	2
Zhu HL [28]	Y	N	N	N	Y	2
ZhangTT [29]	Y	Y	N	N	Y	3
Xie JJ [30]	Y	N	N	N	Y	2
Sun XX [31]	Y	N	N	N	Y	2
Zu YN [32]	Y	N	N	N	Y	2
Dong L [33]	Y	N	N	N	Y	2
Li XB [34]	Y	N	N	N	Y	2
Guo ZY [35]	Y	N	N	N	Y	2
Wang H [36]	Y	N	N	N	Y	2
Zhao N [37]	Y	Y	N	N	Y	3
Wang F [38]	Y	Y	N	N	Y	3

### Clinical Cure Rate

**Figure 2:**
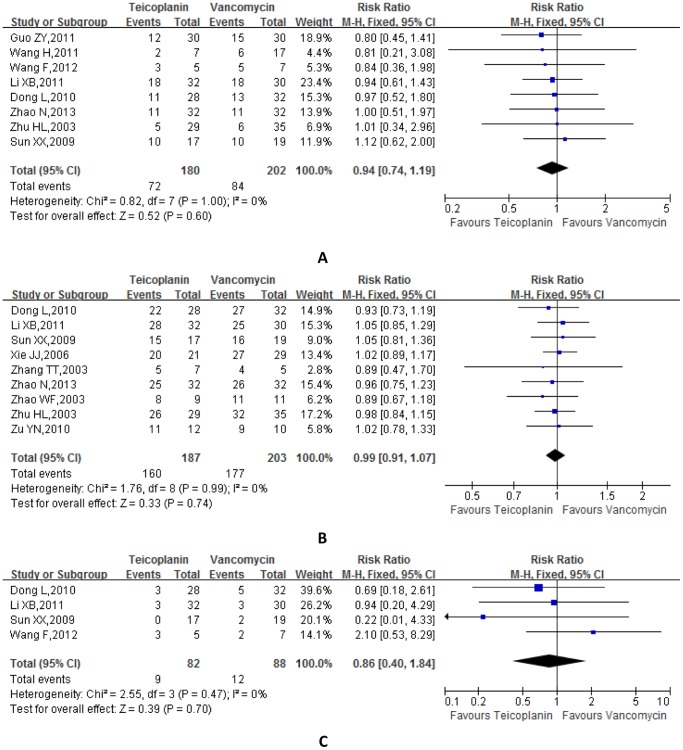
Forest plot of clinical cure rates, microbiological eradication rates and overall adverse events rates. (A) Forest plot for clinical cure rates using fixed-effects model. (B) Forest plot for microbiological eradication rates using fixed-effects model. (C) Forest plot for overall adverse events rates using fixed-effects model.

Eight studies containing the comparison of clinical cure rate (Figure 2A). The heterogeneity was not obvious (P = 1.00 and I^2^ = 0%), so the fixed effect model was applied. The meta-analysis results showed that there was no difference of clinical cure rate between teicoplanin and vancomycin treatment groups (teicoplanin vs vancomycin, RR = 0.94, 95% CI: 0.74∼1.19, P = 0.60).

### Microbiological Eradication Rate

Nine studies involved the comparison of microbiological eradication rate (Figure 2B). The heterogeneity was not obvious (P = 0.99 and I^2^ = 0%), so the fixed effect model was used. The meta-analysis results indicated that the clinical cure rate of teicoplanin was not apparently different from that of vancomycin (RR = 0.99, 95% CI: 0.91∼1.07, P = 0.74).

### Overall Adverse Events Rate

Eight studies have performed the comparison of overall clinical cure rate (Figure 2C). The heterogeneity is not obvious (P = 0.47 and I^2^ = 0%), hence the fixed effect model was employed. The meta-analysis results showed that the overall adverse event rate of teicoplanin is not much distinct from that of vancomycin (RR = 0.86, 95% CI: 0.40∼1.84, P = 0.70). Specifically, several adverse effects were reported in teicoplanin or vancomycin group: liver dysfuncton, leucopenia, nephrotoxicity, red man syndrome, neurologic abnormality, rash and diarrhea. In the vancomycin group, there were three cases of liver dysfunction, one case of leucopenia, five cases of nephrotoxicity, two cases of red man syndrome and one case of rash. However, in the teicoplanin group, there were two cases of liver dysfunction, one case of leucopenia, four cases of nephrotoxity, one case of diarrhea and one case with neurologic abnormality.

### Publication Bias

**Figure 3:**
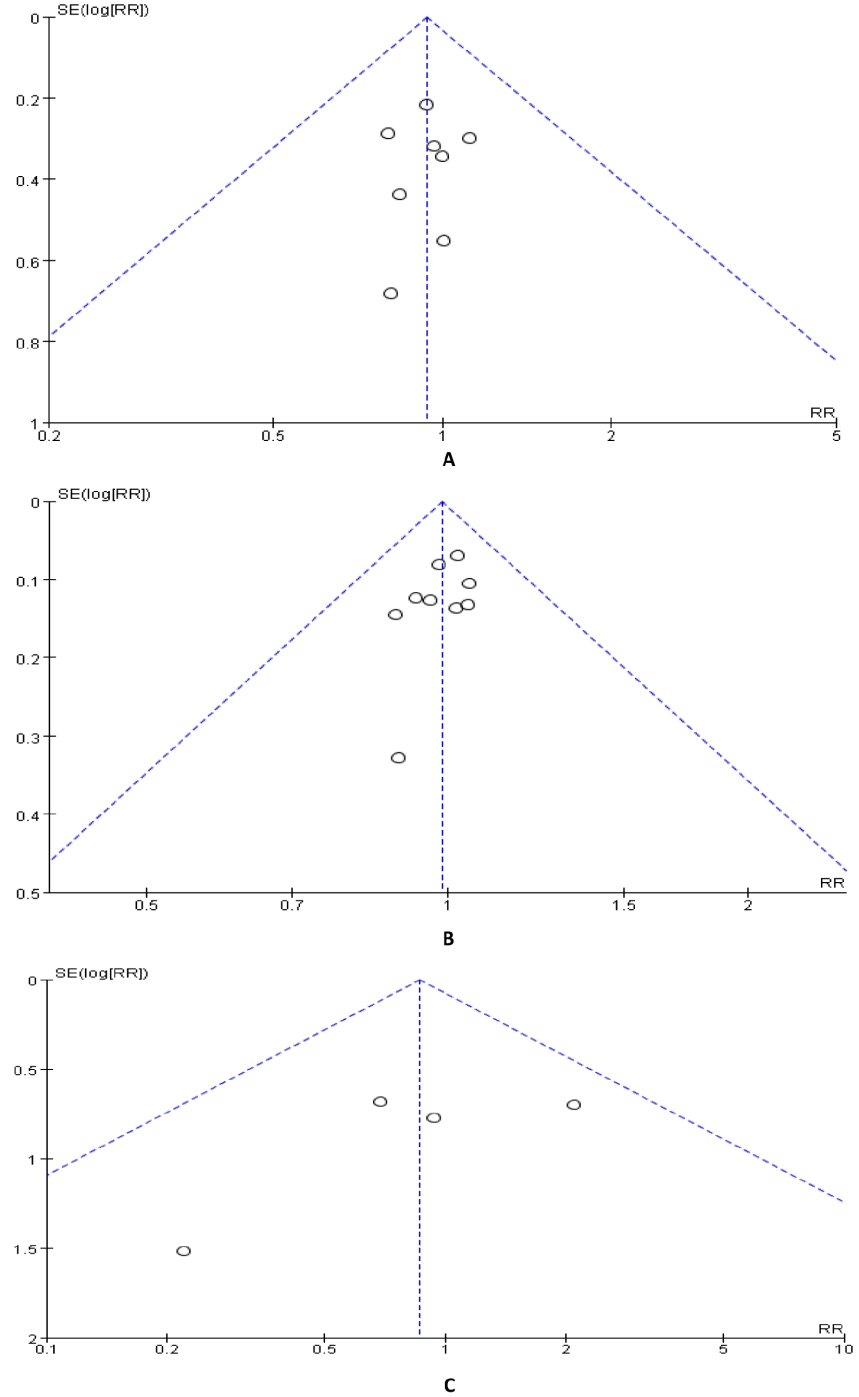
Funnel plot of clinical cure rates, microbiological eradication rates and overall adverse events rates. (A) Funnel plot of clinical cure rates (B)Funnel plot of microbiological eradication rates. (C) Funnel plot of overall adverse events rates.

Funnel plots for clinical cure rate, microbiological eradication rate as well as adverse event rate were displayed. According to the funnel plot, the studies were within the confidential intervals and the shapes of the funnel plots did not reveal any evidence of obvious asymmetry (Shown in Figure 3). However, the numbers of studies were small, so Begger’s test and Egger’s test were performed to further evaluate quantitatively the publication biases. According to the results, all the *p* values of Begger’s test and Egger’s test were above 0.1 (Shown in Table 3). Therefore, there was no strong evidence of publication bias and the results were reliable.

**Table 3:** Outcomes of Begg’s test and Egger’s test.

	Begger’s test		Egger’s test	
	Z value	P value	t value	P value
CC	0.12	1.000	−0.30	0.773
ME	0.52	0.602	−1.00	0.352
AE	0.00	1.000	−0.03	0.983

### Sensitivity Analysis

**Figure 4:**
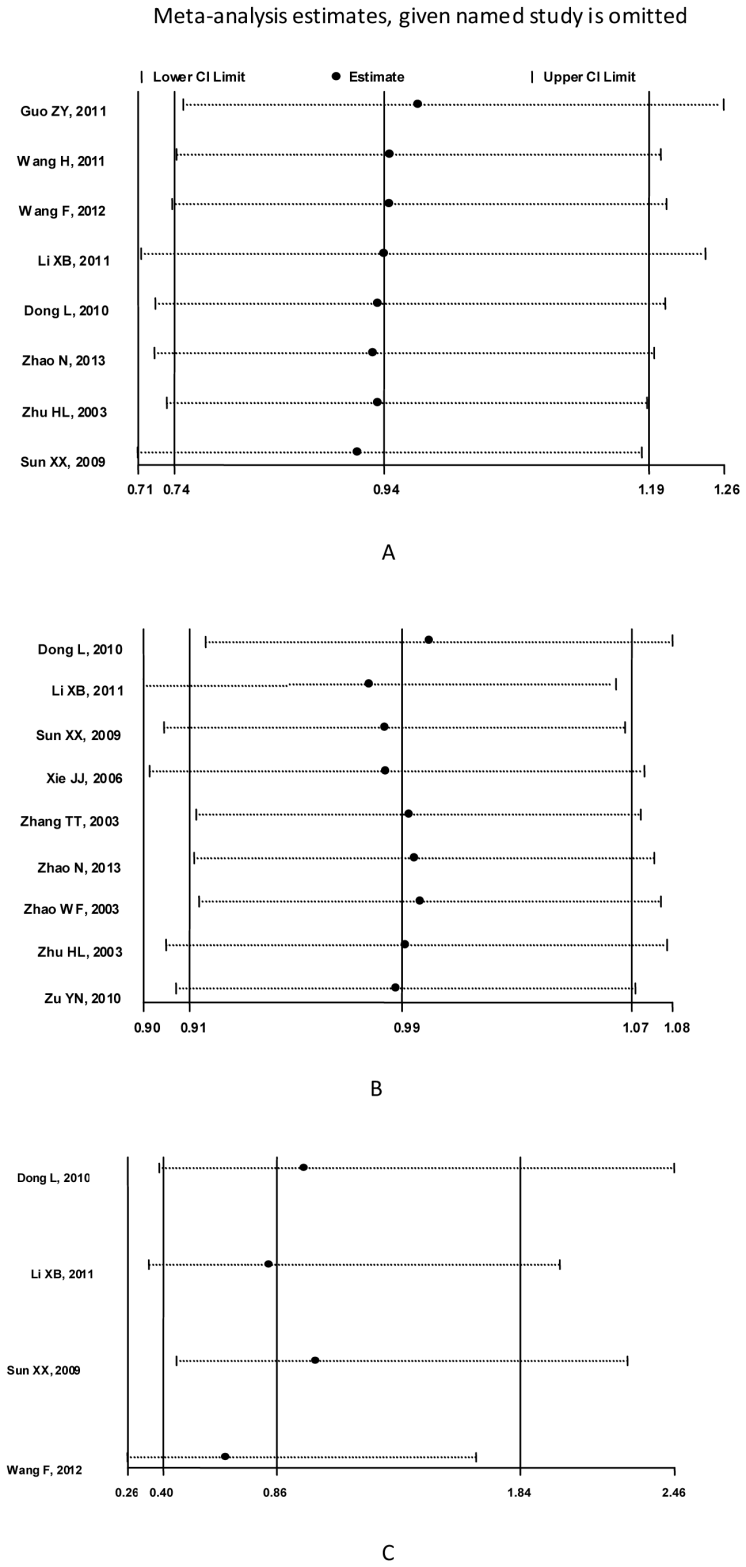
Sensitivity analysis of clinical cure rates, microbiological eradication rates and adverse events rates. Results were computed by omitting each study in turn. The two ends of the dotted lines represent the 95% CI. (A) Sensitivity analysis of clinical cure rates using fixed-effect model. (B) Sensitivity analysis of microbiological rates using fixed-effect model. (C) Sensitivity analysis of adverse events rates using fixed-effect model.

To evaluate the contribution of a single study on the overall pooled RRs and 95% confidential internals, we performed sensitivity analysis by omitting individual studies one by one. The sensitivity analysis indicated that none of the individual studies greatly influenced the overall pooled RR. The leave-one-out RRs estimate ranged from 0.916 (0.710∼1.181) to 0.973 (0.752∼1.258) for clinical cure rate, 0.975 (0.895∼1.062) to 0.996 (0.917∼1.082) for microbiological eradication rate, and 0.654(0.257∼1.664) to 1.018(0.456∼2.276) for overall adverse events rate, respectively, suggesting that the results were consistent (Figure 4).

## Discussion

Teicoplanin and vancomycin, both belong to glycopeptides class, share similar mechanism of antimicrobial activity by binding to the D-alanyl-D-alanine residue of murein monomer and then blocking the biosynthesis of bacterial cell wall. While an experiment of a VSSA strain conducted by Hanaki et al. demonstrated that the over-expression of PBP2’ (penicillin-binding protein 2′) does lead to slightly rise of vancomycin MIC (from 1 to 2 mg/L), whereas that of teicoplanin increased significantly (from 2 to 8 mg/L) [39]. According to relevant study, the over-expression of PBP2’ may bring in the increased rate of cross-linking of cell-wall peptidoglycan instead of thickening of the cell wall. This indicates that there is a distinction in antimicrobial mechanism of action: teicoplanin inhibits more transpeptidation while vancomycin is more inclined to inhibit transglycosylation [9]. In addition, vancomycin and teicoplanin belong to different side-chain linkage patterns and carbohydrate groups [40]. There is no information on the correlation between antimicrobial activities and different chemical structures while it may still lead to different efficacy and safety in MRSA patients; thus, a meta-analysis was conducted.

The results of the meta-analysis indicate that the efficacy of teicoplanin is as effective as that of vancomycin for the treatment of Chinese patients with MRSA infections. The conclusion is based on both the comparison of clinical cure rate as well as microbiological eradication rate. Similarly, teicoplanin has a similar rate of overall adverse events as with vancomycin. We did not compare each single type of adverse event due to the limited sample size. We calculated the Q statistic in order to assess the heterogeneity, and the results are satisfactory for the little implication of heterogeneity. Therefore, any stratified assessments on the source of heterogeneity couldn’t be conducted. Furthermore, the reliability and stability of the studies were tested by the sensitivity analysis, and the results have proved to be stable and reliable in that the effect estimates did not change significantly after in turn removing each single study. To the best of our knowledge, our meta-analysis is the first study to comprehensively evaluate the efficacy and safety of teicoplanin and vancomycin used for treatment of Chinese MRSA patients. A meta- analysis had compared the mortality and adverse events of teicoplanin and vancomycin among MRSA patients, and the results also showed that teicoplanin was as effective as vancomycin with less adverse events [41].

The meta-analysis has some merits. Firstly, all of the included studies are RCTs that have provided comparable and adequate baseline information between the two groups. Secondly, all the subjects are Chinese patients, thus ruling out the impact of ethnicity, which was thought to be a major potential cofounder. Thirdly, the studies are published between 2002 and 2013, which are representative of the safety and efficacy of vancomycin vs teicoplanin in these years. Fourthly, the heterogeneity between the studies is quite small and there was no strong evidence of publication bias and unstable results, thus demonstrating that the outcomes were both reliable and stable. Finally, the included studies were dispersed in ten cities of eight provinces, four cities lie in North China and six cities lie in South China, which can comprehensively represent the population distribution of the whole country.

Nevertheless, our meta-analysis also bears some limitations and drawbacks. Firstly, the number of the participate subjects and articles are small. This may decrease the power of our analysis. Secondly, the qualities of the included studies are relatively poor because the Jadad scores of them are between 2 and 3. Thirdly, we cannot take account of the influence of other factors such as the original health situations of the patients, the quality care of the doctors and hospitals for the authors of the included articles failed to offer such information. Finally, we are ineligible to collect the data from unpublished articles, therefore we cannot speculate whether there are similar results or not.

In conclusion, our study demonstrates that teicoplanin and vancomycin showed similar efficacy and safety in the treatments of MRSA in Chinese patients. Teicoplanin could be used as an alternative antibiotic for vancomycin when vancomycin is not effective. More high-quality RCTs are needed to evaluate the two antibiotics in a more accurate way.

## Supporting Information

Checklist S1 **PRISMA checklist of this meta-analysis.**
(DOC)
